# Excavation of acoustic nanostructures biosynthesis gene clusters by combinatorial strategy

**DOI:** 10.1007/s44307-025-00069-5

**Published:** 2025-05-15

**Authors:** Wei Liu, Tingting Liu, Shenxi Huang, Fei Yan, Jian-Zhong Liu

**Affiliations:** 1 State Key Laboratory of Biocontrol, School of Life Sciences, Sun Yat-Sen University, Guangzhou, 510275 People’s Republic of China; 2 Department of Ultrasound, The Second People’s Hospital of Shenzhen, The First Affiliated Hospital of Shenzhen University, Shenzhen, 518061 China; 3 CAS Key Laboratory of Quantitative Engineering Biology, Shenzhen Institute of Synthetic Biology, Shenzhen Institutes of Advanced Technology, Chinese Academy of Sciences, Shenzhen, 518055 China

**Keywords:** Acoustic nanostructures, Gas vesicles, Ultrasound imaging, Combinatorial biosynthesis strategy, *Escherichia coli*, Site-saturation mutagenesis

## Abstract

Gas vesicles (GVs) produced by microorganisms are genetically engineered, air-filled protein nanostructures that have widespread applications in ultrasound imaging and ultrasound-mediated drug delivery. However, constrained by the shape and size, most of them are difficult to be imaged by clinical ultrasound machines, which limits their biomedical applications. Here, we constructed a hybrid gene cluster of the structural gene cluster from *Serratia* sp. ATCC 39006 and the accessory gene cluster from *Bacillus megaterium* in *Escherichia coli* to synthesize a novel gene-encoded gas vesicle with a width of approximately 70 nm and a length of about 100 nm, using a synthetic biology strategy, termed as ARG_S1B_. This new type of GVs can be stably produced in bacteria and is able to be imaged by clinical ultrasound machines in vivo and in vitro. Furthermore, the novel nanostructure can be easily engineered for different particle sizes through point saturation mutation, expanding the sources of GVs and providing new insights into the biosynthesis mechanism of GVs.

**Supplementary Information:**

The online version contains supplementary material available at 10.1007/s44307-025-00069-5.

## Introduction

Ultrasound, one of the most recognized medical imaging modalities, has been widely used in biomedicine, particularly in obstetrics and internal medicine (Pfeifer 2022). With the development of ultrasound contrast agents, ultrasound imaging is playing more and more important roles in disease diagnosis, therapeutic evaluation and fundamental biological researches such as in vivo tracking of stem cells. Thanks to their enhanced cavitation effects, ultrasound contrast agents may amplify the biological effects of ultrasound, widening its therapeutic applications in thrombolysis (Wu et al. 2017), targeted drug delivery (Bez et al. 2019; Walker et al. 2019; Wang et al. 2022; Yong et al. 2022) and high-intensity focused ultrasound (HIFU) (Bar-Zion et al. 2021; Yang et al. 2024) etc. Gas vesicles (GVs), a type of gas-filled protein nanostructure composed of an amphipathic protein shell with a ribbed ultrastructure primarily encoded by the structural proteins GvpA and GvpC, have been demonstrated to produce acoustic signals and can function as acoustic report genes or probes for ultrasound imaging (Lakshmanan et al. 2016).

Since the discovery of gas vesicles in cyanobacteria a century ago (Cai et al. 2020; Huber et al. 2024), GVs have been identified in many photosynthetic cyanobacteria including *Anabaena flos-aquae*, *Planktothrix* and *Microcystis aeruginosa *etc., allowing them to float and remain close to the surface in order to convert light into chemical energy (Bourdeau et al. 2018; Dutka et al. 2023; Jung et al. 2004). Later, researchers discovered that a wide variety of microorganisms, including heterotrophic bacteria, *Bacillus megaterium*, *Archaea*, *Actinomycetes*, *Haloferax mediterranei*, *Haloquadratum walsbyi* and *Rhodobacter sphaeroides *etc*.* (Bolhuis et al. 2004; Burns et al. 2004, 2007 Zimmermann et al. 2003) are also capable of producing GVs. Although a large number of species were identified to be able to produce GVs, these naturally biosynthesized GVs have fixed size and shape, typically with widths of 45—250 nm and lengths of 100—800 nm, depending on their genetic origins (Pfeifer 2012). Up to now, the most researches are major in modification and application of GVs encoded by ARG1, acoustic reporter genes, which initiatively created by Shapiro’s group using hybrid gene clusters and successfully applied for noninvasive imaging in mammalian hosts (Bourdeau et al. 2018). Besides that, all the researchers focus on the synthesis and modified application of the GVs encoding by the natural host like *halophilic archaeon* (Pfeifer et al. 2002; Tayier et al. 2019) and blue-green algae (Long et al. 2023; Pfeifer 2012). Lu’s group engineered GV mutants to obtain 50-nm stable, free-floating, gas-filled protein nanostructures and achieved lymphatic tissue infiltration (Shen et al. 2023). The diversity of GVs is extremely scarce because the heterologous expression of GVs synthesis gene clusters is difficult, which primarily attribute to the inherently weak acoustic scattering and buoyancy of the small gas vesicles produced by their hosts. These obstacles greatly limit the establishment of high-throughput screening methods and characterization approaches, which have led to a noteworthy workload in the search and identification of novel gene-encoding gas vesicles.

**Fig. 1:**
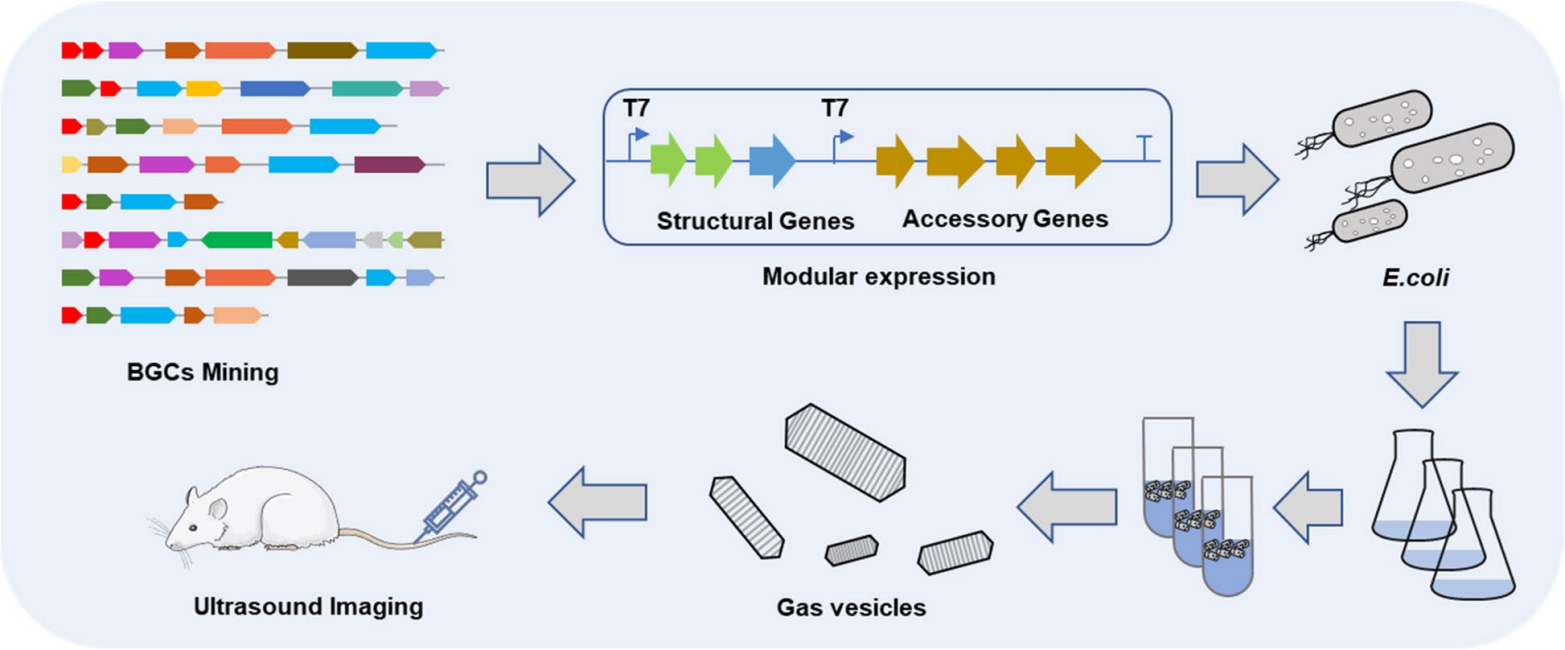
Workflow for mining GVs biosynthetic gene clusters (BGCs) by combinatorial biosynthesis technique

Here we constructed a new class of engineered gas-filled nanostructure with great ultrasound response using combinatorial biosynthesis techniques which are mainly applied to drug discovery (Li et al. 2009; Sun et al. 2015; Weissman et al. 2005) and the biosynthesis of natural products (Skellam et al. 2024; W. Zhang et al. 2008; Zhou et al. 2008). We screened gene clusters capable of synthesizing excellent GVs by summarizing and organizing relevant reviews and literature. Then, in order to enrich the species sources, we selected widely studied GV synthesis gene clusters from sources such as archaea, actinomycetes, cyanobacteria, and proteobacteria for subsequent combinatorial biological synthesis strategies, and ultimately obtained six gene clusters for the gene library. Afterward, we separated the gene clusters into two categories, structural genes and accessory genes, and then freely combined these two groups of gene clusters by cloning expression under T7 promoter to test the ability of GVs synthesis. Obtaining the top layer cells to ascertain the true existence of gas vesicles by Transmission electron microscopy (TEM) (Lakshmanan et al. 2017) after placing them stably for 48 h and comparing the suspension bacterial cells in conical tubes. Fortunately, we screened a novel gene cluster that could stably express gas vesicles in bacterial cells, and its acoustic contrast imaging performance was further evaluated in vitro and in vivo (Fig. 1).

## Materials and methods

### Bacterial plasmids construction and protein expression

The sequence of BGCs were synthesized by GENEWIZ, Guangzhou, China. Each BGC was divided into two modules and cloned into pETDuet plasmid downstream of T7 promoter respectively. All constructs were made via restriction cloning using enzymes from Thermo Scientific or homologous recombination using One Step Cloning Kit from Vazyme. The mutations of the GvpA of ARG_S1B_ were using Taq DNA Polymerase from Vazyme and sequenced by Sangon (Guangzhou, China). Purified plasmids were transformed into *E. coli* BL21 (AI) competent cells (Thermo Fisher Scientific) and plated on Luria broth (LB)-agar medium containing 100 μg mL^−1^ ampicillin for 16 h at 37 °C. Single colonies were isolated and grown in 5 mL LB medium containing 100 μg mL^−1^ ampicillin and 1% glucose at 37 °C for overnight. The overnight cultures were inoculated at 1:100 dilutions into 40 mL LB medium supplemented with 100 μg mL^−1^ ampicillin and 0.2% glucose for large-scale amplification. For induced GVs biosynthesis, *E. coli* cells were grown at 37 °C with shaking until OD_600_ = 0.5, and then induced with 0.8 mM IPTG and 1% L-arabinose at 37 °C for 72 h.

### Flotation assay

The GV biosynthesis was carried out by the above method. 10 mL of the bacteria were put into 50 ml sterilized centrifuge tubes. After 48 h, 1 ml below the surface was taken and OD 600 was measured.

### Gas vesicle preparation

Gas vesicles were produced in *E. coli* BL21 (AI) using a modified preparation protocol (Lakshmanan et al. 2017). GVs were purified with 3 steps of 1 h centrifugation at 600 g at 4 °C, each time transferring the floating phase into PBS. GVs concentration was determined with absorbance measurement at 500 nm.

### Hydrodynamic size and zeta potential measurements

The particle size and zeta potential of GVs were measured using a Zetasizer analyzer (Zetasizer NanoS90, Malvern, Worcestershire, UK).

### TEM sample preparation and imaging

GVs were washed three times with distilled water before TEM sample preparation. Samples were deposited on Formvar/carbon 200 mesh grids (Ted Pella) that were rendered hydrophilic by glow discharging before (PELCO 91000). And then 2% PTA (phosphotungstic acid) was added for staining. The samples were then imaged on JEM-1400 Flash. Images were processed with RADIUS and ImageJ.

### In vitro ultrasound imaging

Ultrasound imaging phantoms were prepared by melting 1% w/v agarose in distilled water and casting wells with EP tubes. GVs at different concentrations were added into 1% agarose phantom wells. The ultrasound imaging performance of GVs was detected by using an ultrasound device with a linear array transducer (Mindray Resona 9 T, Mindray, Shenzhen, China). Imaging parameters were kept constant during all imaging sessions (frequency: 7.1 MHz, depth: 2.5, mechanical index = 0.320, frame rate: 10, dynamic range: 115, contrast gain 70 dB).

### In vivo ultrasound imaging

All animal experiments were performed on 5–6 weeks BALB/c female mice (≈20 g, purchased from Beijing Vital River Laboratory Animal Technology Co., Ltd) and approved by the Institutional Animal Care and Use Committee (IACUC) of the Animal Experiment Center of Shenzhen Institutes of Advanced Technology, Chinese Academy of Sciences (SIAT-IACUC-210831-HCS-YF-A2043). In vivo ultrasound imaging was performed under isoflurane anesthesia, and all efforts were made to minimize pain, discomfort, and suffering. As for liver imaging, we randomly injected GVs (150 μL, OD500 = 3.0) into mice via the tail vein to minimize bias to ensure the reliability of the experimental results. GVs were injected via the tail vein by a single bolus injection and imaged using a L11-3U line array transducer ultrasound diagnostic equipment (Mindray Resona 9 T, Mindray, Shenzhen, China). In vivo, ultrasound imaging parameters (acoustic power = 10.96%, mechanical index = 0.228, transducer transmit frequency = 7.1 MHz, frame rate: 10, contrast gain = 70 dB, dynamic range = 115) were consistent across experimental conditions. The perfusion area was defined as the region of interest (ROI). The acoustic signal intensities of ROI were quantitatively analyzed using ImageJ software.

### Statistical analysis

GraphPad Prism 10.3.1 software was used for statistical analysis. Statistical analysis was performed using unpaired Student’s t-test. Asterisks represent statistical significance by two-tailed (*p < 0.05; **p < 0.01; ***p < 0.001; ****p < 0.0001; ns, not significant).

## Results and discussion

### Excavation of the novel GVs by gene cluster hybridization

GVs have been identified in a wide range of bacterial and archaeal phyla, exhibiting variations in size and type across different species, which are mainly determined by the sequence of GV operons. The most commonly observed have a spindle body and cylindrical shape (Dutka et al. 2023; Huber et al. 2023), with particle sizes ranging from 30 to 500 nm, with varying degrees of ultrasonic signal depending on diameter and shape.

**Fig. 2:**
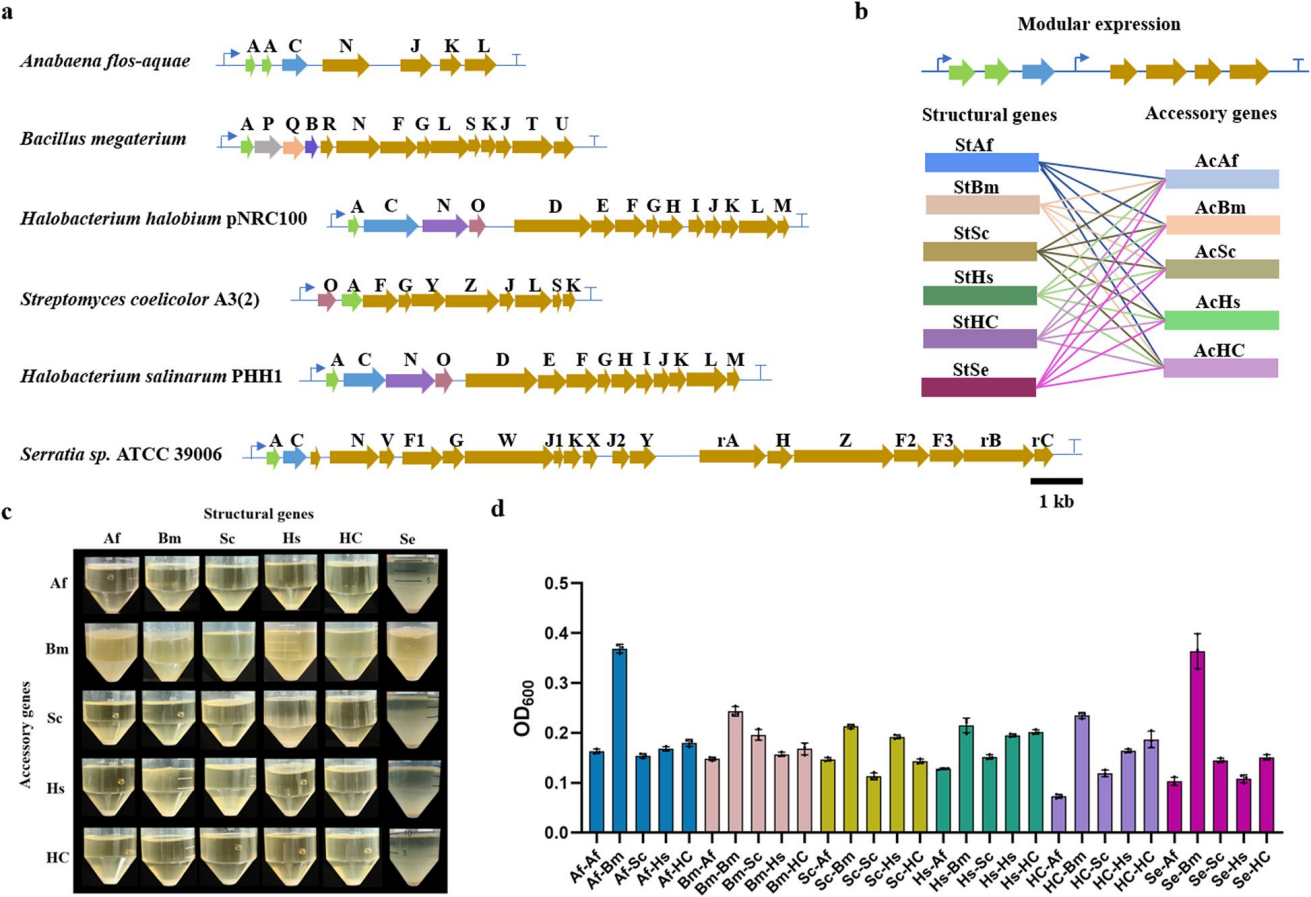
**a** The final BGCs after modular analysis in this study. **b** The combination of different gene clusters. (St, structural gene; Ac, accessory genes; Af, *Anabaena flos-aquae*, Bm, *Bacillus megaterium*, Sc, *Streptomyces coelicolor* A3(2), Hs, *Halobacterium* sp. NRC-1 pNRC100, HC, *Halobacterium salinarium* C-GVP, Se, *Serratia* sp. ATCC 39006). **c** Flotation assay results after stewing 48 h. **d** Average OD_600_ in Flotation Assay, three biological replicates

**Fig. 3:**
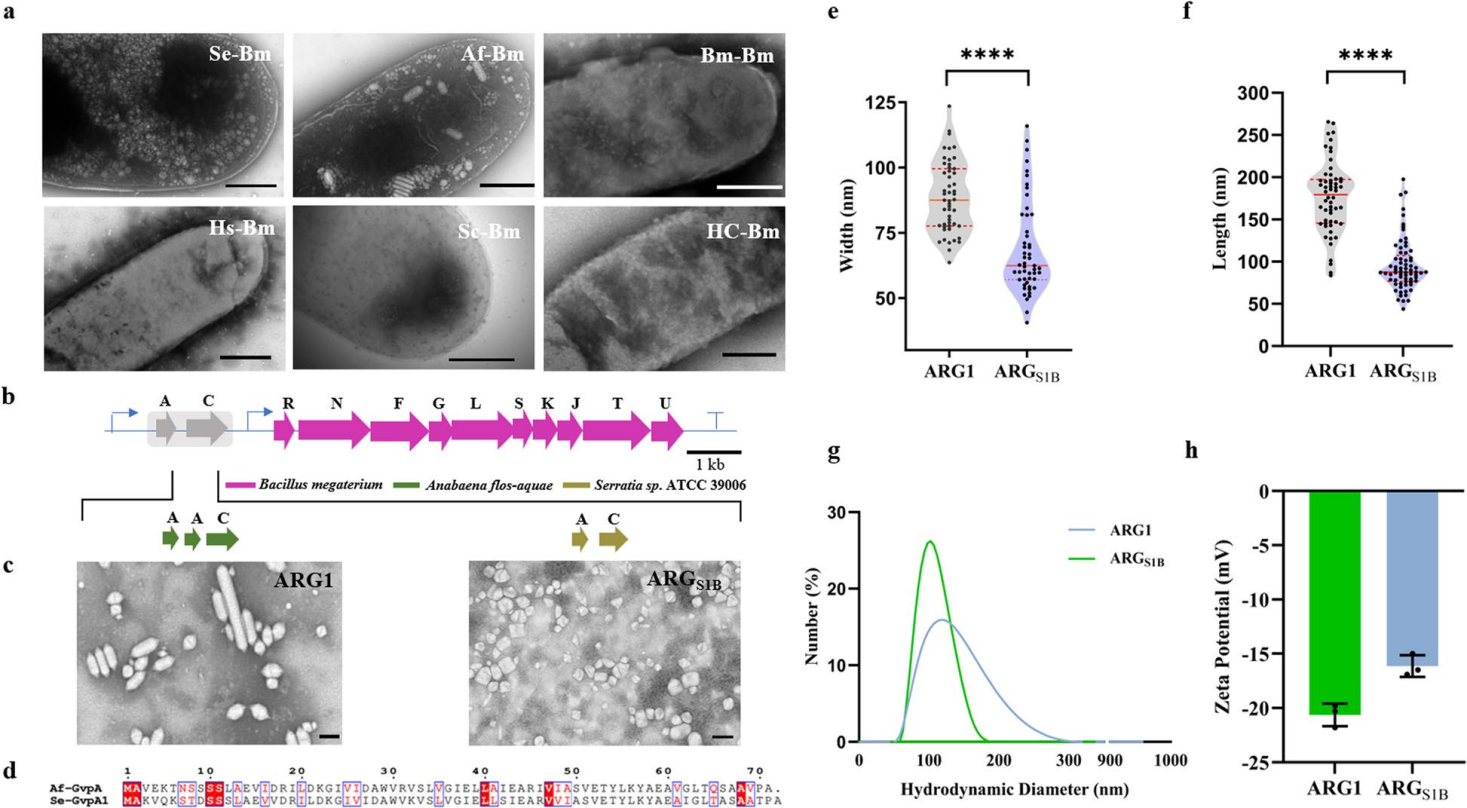
Characterization results of ARG_S1B_ in vitro. **a** TEM images of *E. coli* cells expressing different gene combinations (Scale bars, 500 nm), see also Figure S1. **b** Organization of GVs biosynthetic gene clusters; the region highlighted in grey was varied. **c** TEM images of ARG_S1B_ and ARG1 (Scale bars, 200 nm). **d** Sequence alignment of GvpA from *A. flos-aquae* and *Serratia sp.* ATCC 39006. **e**, **f** Violin plots showing the particle width and length. Red dashed horizontal lines indicate the interquartile range and a red solid horizontal line indicates medians; significance levels, ****p < 0.0001. **g** Hydrodynamic diameters of ARG_S1B_ and ARG1. **h** Zeta potential analysis was conducted with *N* = 3 biological replicates

Inspired by the hybrid gas vesicles ARG1 from *B. megaterium* and *A. flos-aquae* showing an ultrasound imaging properties (Bourdeau et al. 2018), we hypothesized that the combination of various structural and accessory genes could potentially lead to the formation of novel types of gas vesicles. To explore this possibility, we proposed a gene combination strategy to screen for new gene clusters for GVs synthesis. We first sought the gene clusters which had been reported that could synthesize the gas vesicles in the host bacterium like *Archaea*, *Cyanobacteria *etc. Comprehensive consideration of the species category, the particle diameter and the length of gene cluster which decided the difficulty level of gene synthesis, the gas vesicle clusters from *A. flos-aquae* (GenBank: U17109.1), *B. megaterium* (GenBank: AF053765.1), *Halobacterium* sp. NRC-1 pNRC100 (GenBank: M58557.1), *Halobacterium salinarium* C-GVP (GenBank: X64730.1), *Streptomyces coelicolor* A3(2) (GenBank: NC_003888.3), and additional *Serratia* sp. ATCC 39006 (GenBank: CP025084.1) were selected to combine (Fig. 2a). The structural gene cluster from different microorganisms under the control of T7 promoter was paired with the accessory gene cluster from different microorganisms under the control of T7 promoter. In this case, we obtained a total of 30 different combinations of GVs synthesis gene clusters for the library. These engineered gene clusters were introduced in *E. coli* (AI) for heterologous expression (Fig. 2b). After induced fermentation using IPTG and L-arabinose, stewing and then watching the biomass of floating bacterium to estimate the expression level of gas vesicles preliminarily. As shown in Fig. 2c, the combinations containing accessory genes from *B. megaterium* had significantly higher floating bacterial cells than other combinations. However, most of remaining heterozygous gene clusters could not float indicating that it did not synthetize the GVs. What’s more, it’s obviously that two combinations had significantly high biomass which means they may have great GVs expression (Fig. 2d, Table S1). Fortunately, we ultimately discovered the two combinations: the structural gene cluster from *Serratia* sp. ATCC 39006 and the accessory gene cluster from *B. megaterium*, and the structural gene cluster from *A. flos-aquae* and the accessory gene cluster from *B. megaterium*, which lead to the formation of gas vesicles determined by TEM (Fig. 3a, Figure S1). Notably, the second combination corresponds to the same gene cluster as ARG1, as previously reported by Shapiro et al. (Bourdeau et al. 2018). Therefore, the first hybrid gene cluster (the structural gene cluster from *Serratia* sp. ATCC 39006 and the accessory gene cluster from *B. megaterium*) was designated as ARG_S1B_ for future experiments in this study. In order to better characterize the performance of the GVs, we then optimized the microbial synthesis conditions to increase the yield of the GVs. When the fermentation time was extended to 3 days, there was a significant increase in cell growth and the number of floating bacterial cells (Figures S2, S4). Subsequently, the fermentation temperature was further increased to 37 ℃, and the cell growth and floating bacterial cell numbers were further improved (Figures S3, S4), indicating that fermentation at 37 ℃ for 3 days is more conducive to the synthesis of GVs (Figure S4).

### Nanoparticle characterization of ARG_S1B_

As the TEM showed (Fig. 3c), ARG1 were small, bicone-shaped gas vesicles, while ARG_S1B_ tend to be spherical shape with smaller diameter. Both of them have a high degree of conservatism in the sequence of GvpA, which might be the reason that this combination can form GVs (Fig. 3b, d). Then we established the dimensions under TEM preliminarily by manual statistics (Dutka et al. 2021), which showed that the mean length and width of ARG_S1B_ was 95.50 ± 30.96 nm and 68.75 ± 17.83 nm, while ARG1 was 176.50 ± 42.59 nm and 88.72 ± 13.57 nm respectively (Fig. 3e, f). In order to systematically compare the diameter, we proceeded to measure the hydrodynamic diameter of GVs in a hydrated condition by dynamic light scattering (DLS), which better predicts their behavior in biomedical applications. The results showed that the hydrodynamic diameter of ARG_S1B_ was 106.90 ± 8.91 nm, which was slightly smaller than the ARG1 (122.57 ± 7.13 nm) (Fig. 3g). Then, we test the zeta potential, a measure that can be used to draw conclusions about the electrostatic stabilization of droplets and particles, reflecting the strength of mutual repulsion or attraction between particles (Manciu et al. 2017; Souza et al. 2023; Teeranachaideekul et al. 2007). As we know, the higher the absolute value (positive or negative) of Zeta potential, the more stable the system, that is, dissolution or dispersion can resist aggregation. And the absolute value of Zeta potential represents its stability level. The result demonstrated that both of two types of gas vesicles have negatively charged surfaces, and become unstable (the absolute value of Zeta potential represents between 10 and 30). The ARG_S1B_ has less surface charge may be due to the smaller particle size containing a smaller surface area (Fig. 3h).

### Verification of acoustic activity

**Fig. 4:**
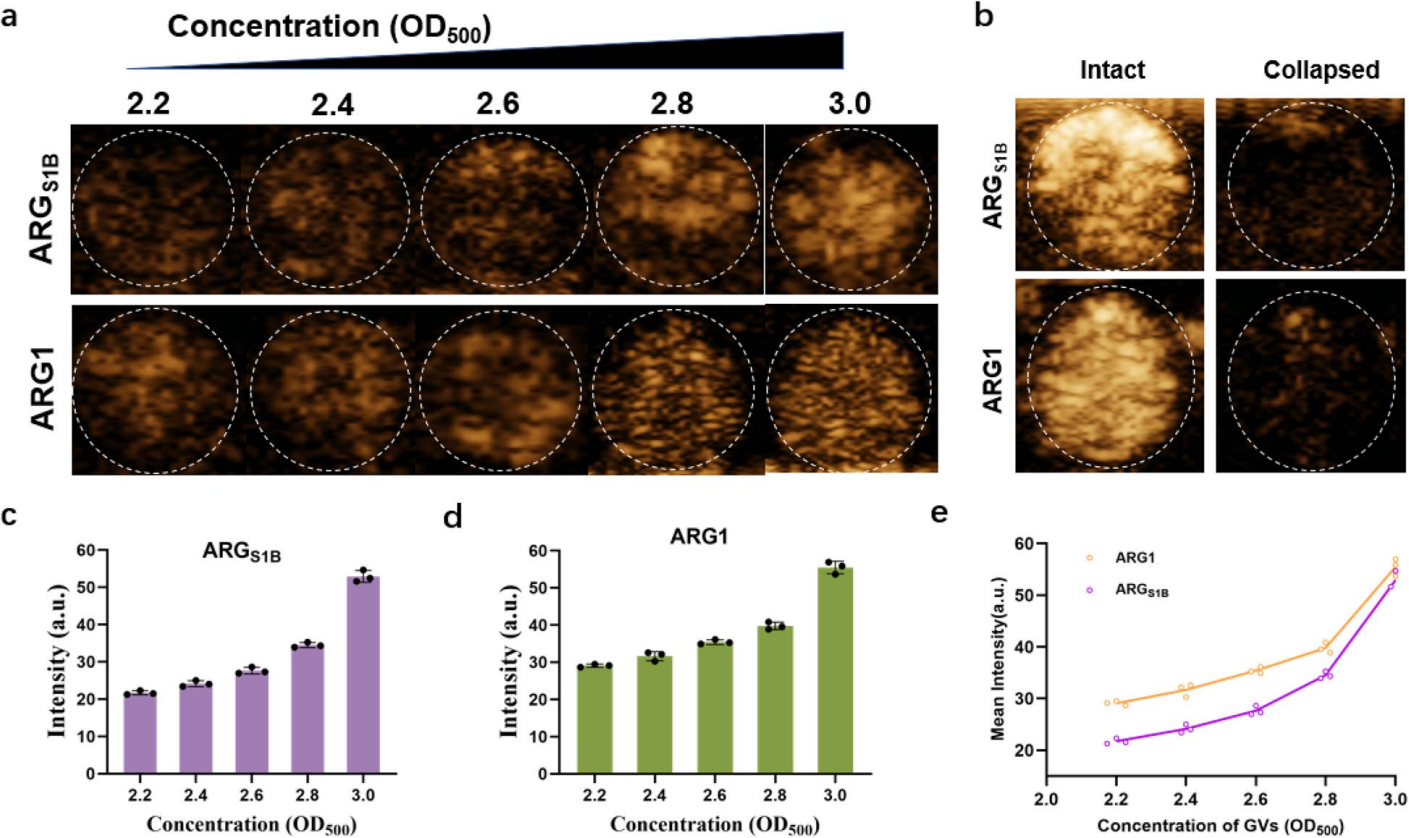
Characterization of ARG_S1B_ in vitro. **a** The ultrasound image of a phantom containing serial dilutions of GVs with different OD_500_. **b** The ‘BURST’ images of a phantom containing GVs of ARG_S1B_ (OD_500_ = 3.2) and ARG1 (OD_500_ = 2.7), before and after acoustic collapse. **c**, **d** Quantification of the signal intensity target ROIs from images in (a) and other replicates. **e** Mean ultrasound contrast from ARG1 and ARG_S1B_ at various GV densities. Error bars representing mean ± SD for *N* = 3 replicates

Previous research had confirmed that the floating of the bacterium was caused by the gas-filled nanostructure inside (Bouma-Gregson et al. 2017; Hill et al. 2020). As for medicine used in the clinic, ultrasound contrast agent must have acoustic activity like ultrasonic fragmentation, imaginable in vivo and in vitro. Thus, we first tested the contrast imaging ability in an agarose phantom using a small animal ultrasound imaging system. In contrast mode, both two gas vesicles can receive acoustic contrast signals. When click on BURST mode (Sawyer et al. 2021), we barely got any signals, which shows that ARG_S1B_ can collapse during high pressure like ARG1 (Fig. 4b). Then, we tested the acoustic contrast signals using gas vesicles with increased concentration (Fig. 4a). When the concentration of gas vesicles achieved OD_500_ = 3.0, the mean signal intensity at the region of interest (ROI) reached 52.935 ± 1.570 (a.u.), while ARG1 was 55.497 ± 1.653 (a.u.) (Fig. 4c, d). The above results showed that ARG_S1B_ had similar ultrasound characterization with ARG1 in vitro (Fig. 4e).

**Fig. 5:**
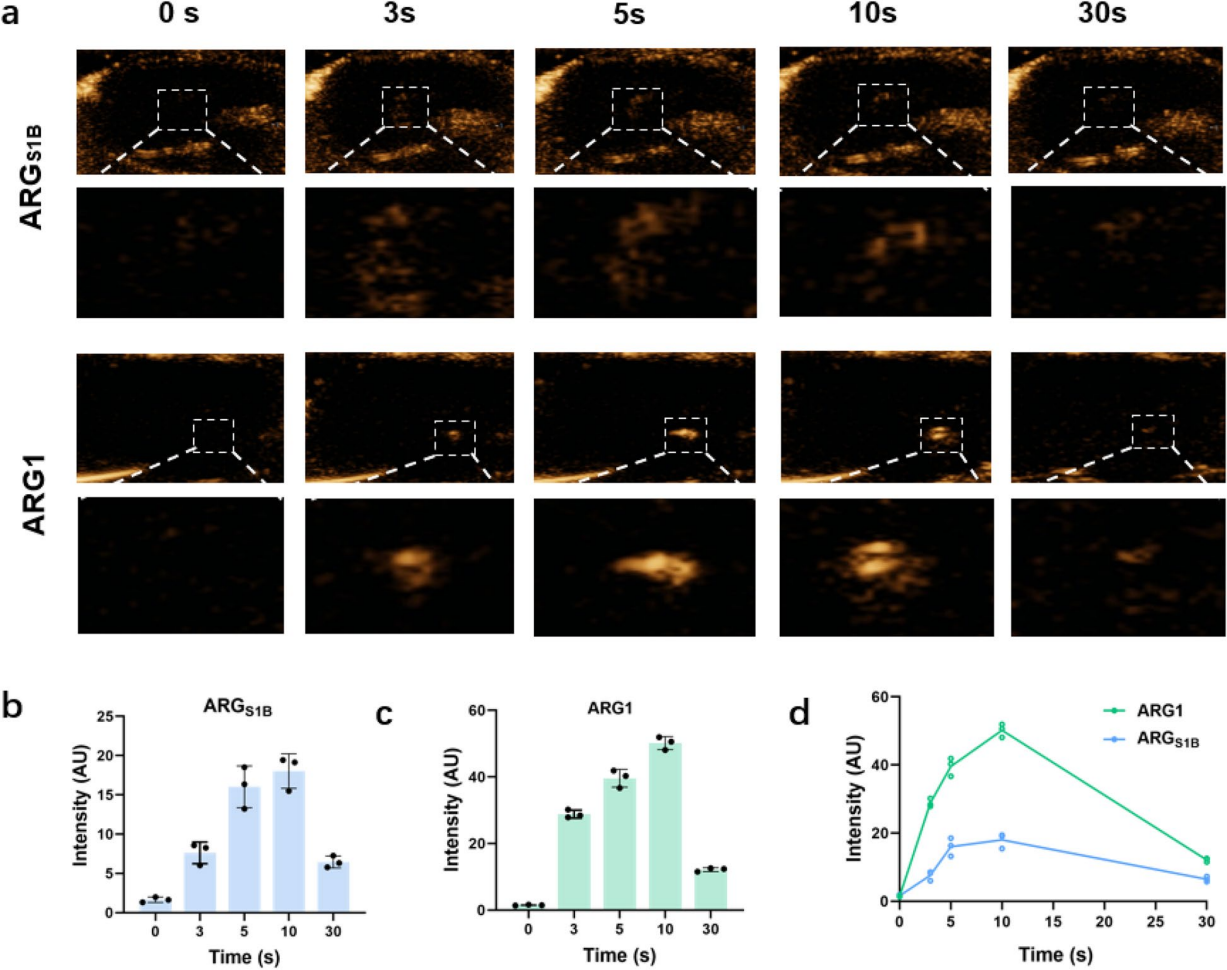
Characterization of ARG_S1B_ in vivo. **a** The ultrasound contrast images of the liver received with ARG_S1B_ or ARG1 at different time points, OD_500_ = 3.0. **b**, **c** Contrast signal intensity in the liver (white-dotted rectangular regions). **d** Mean ultrasound contrast from ARG1 and ARG_S1B_ at different injection time. Error bars representing mean ± SD for *N* = 3 replicates

To further evaluate the contrast imaging capability of ARG_S1B_, we conducted in vivo experiments in healthy mice. We injected the same concentration GVs of ARG_S1B_ and ARG1 into the tail vein of mice and imaged their livers in contrast mode using a small animal ultrasound imaging system (Fig. 5a-c). Comparing the contrast signals at different times after injection, we can significantly find that after 10 s of injection, the signals of ROI (white-dotted rectangular regions) reached the peak value, and after 30 s of injection, the signals of ROI touched bottom (Fig. 5d), which mean the gas vesicles had dispersed in the liver with the flow of blood. The results indicated that the imaging capability of ARG_S1B_ was weaker than that of ARG1, which might be because the particle size of ARG_S1B_ was smaller than ARG1 and had a weaker cavitation effect.

### Genetic engineering alters the particle size of GVs

**Fig. 6:**
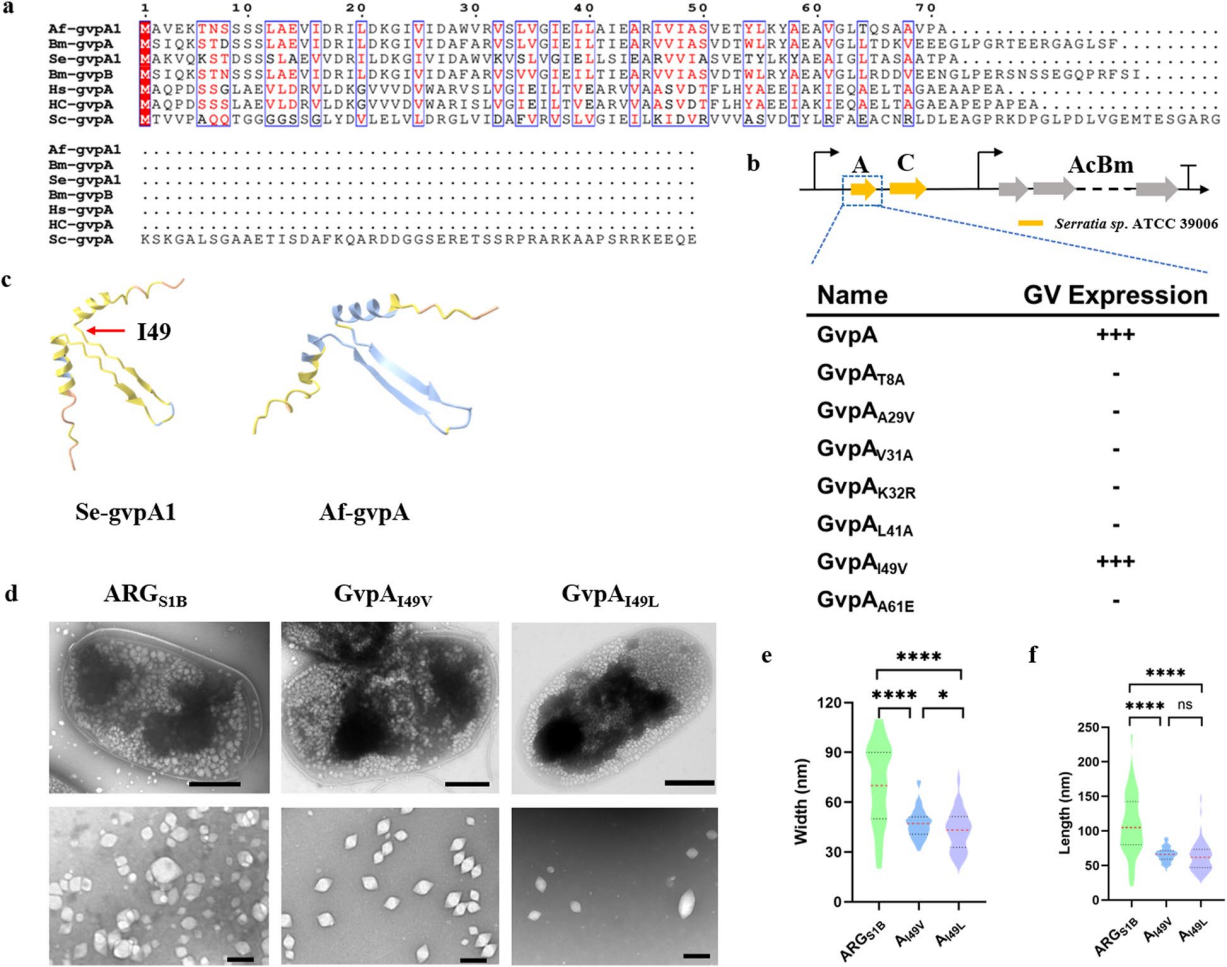
Controllable modification of GVs particle size. **a** Sequence alignment of GvpA derived from different species using ESPript 3.0. **b** Engineered GV mutants. Subscripted names stand for mutations, for example, T8 A. **c** Protein structure prediction of GvpA derived from *Serratia* sp. ATCC 39006 and *A. flos-aquae* using Alpha Fold. **d** TEM images of ARG_S1B_ and GvpA mutations (Scale bars, bacteria, 500 nm; GVs, 200 nm). **e**, **f** Violin plots showing the width and length of particle respectively. Black dashed horizontal lines indicate the interquartile range and a red solid horizontal line indicates medians (significance levels, ****p < 0.0001; ns, not significant)

As we know, the function of proteins depends on their structure. Up to now, we still know a few about protein–protein interaction and the GV assembly in cells. In order to illustrate the reason why only the structure gene derived from *Serratia* sp. ATCC 39006 could form gas vesicles with the accessory genes derived from *B. megaterium*, we conducted multiple sequence alignments which showed all six structure proteins harbored highly conservative areas that represented coil-α-β-β-α-coil motif (Huber et al. 2023), only the N-term and C-term had a significant difference in sequence (Fig. 6a). And the GvpA from *Serratia* sp. ATCC 39006 and *A. flos-aquae* had a high similarity. Furthermore, we attempted to use Alpha Fold to predict the 3D structure (Fig. 6c). We could find that the GvpA from *Serratia* sp. ATCC 39006 and *A. flos-aquae* were highly overlapped in β-sheet structure which was the major components that make up the surface of GVs. And the α helices just lead to steric hindrance with another symmetry α helice according to Huber et al. (Huber et al. 2023). We guessed that may be the reason that only structure protein derived from *Serratia* sp. ATCC 39006 could form the novel gas vesicles similar to ARG1.

Based on the analysis above, we attempted to obtain the specific site which determined the shape and diameter in GvpA, resulting the first set of GV mutants to experience: T8 A, A29 V, V31 A, K32R, L41 A, I49 V and A61E (Fig. 6b). From these designs, we uncovered a genetic variant, I49 V mutation, that had slight floating after centrifugation (Figure S5). And the TEM showed that it’s a novel type GV significantly different from the ARG_S1B_ (Fig. 6d). Remarkably, the width of this GV variant was measured to be 47.16 ± 8.83 nm, and the length was 66.07 ± 10.46 nm (Fig. 6e, f), which significantly reduced the diameter compared to ARG_S1B_. Refer to the cryo-EM structure of GvpA of *B. megaterium* (Shen et al. 2023) which had high sequence homology, we could find that the 49 th amino acid just located at the coil between β2 sheet and α2 helices, which might influence the included angle of these two structures and further change the particle size of GVs.

Having identified the key amino acid, we carried out site-saturation mutagenesis (Sarkar et al. 2022; F. Zhang et al. 2024) to explore other possibilities that could lead to the synthesis of GVs of different sizes and shapes. The result indicated that only I49L variant also had significant GVs synthesis with a similar diameter compared to I49 V according to TEM (Fig. 6d). Taking all these variants together, we could find a commonality that they are the most hydrophobic amino acids among the 20 amino acids, which indicated that they may alter the density by affecting the strength of hydrophobicity rather than changing the included angle between β2 sheet and α2 helices as we guessed before. What’s more, the discovery of this special amino acid site suggests that modifications to other sites may lead to an increase in the size of the GVs particles, demonstrating a certain degree of plasticity.

## Conclusion

In brief, in order to discover more distinctive resources of gene clusters for gas vesicle biosynthesis, we conduct a gene cluster combinatorial strategy to screen and obtain a novel gene cluster, ARG_S1B_, which can achieve great performance in ultrasound imaging in vivo and in vitro. Later on, we use sequence alignment to obtain distinctive variants, which can significantly change the shape and diameter of GVs, exhibiting plasticity in physical properties. The diameter of the GVs is reduced to nearly 50 nm, which can enhance the tissue penetration and can directly access cells in deep tissues (Shen et al. 2023). Remarkably, the emergence of ARG_S1B_ and engineered variants can greatly enrich the existing available contrast agent resources, and provide a brand-new example for the shape modification and genetically engineering, promoting the profound study on the understanding of the molecular mechanism. I The future of ultrasound contrast agents is bound to gradually expand from the field of medical diagnosis to the field of treatment, thanks to the development of synthetic biology and the deep integration of multiple disciplines. Currently, some studies have shown that the cavitation effect of GVs can be used for targeted blasting of tumors, achieving the goal of killing tumors (Bar-Zion et al. 2021). However, the prerequisite for expanding the application is the need for a sufficient variety of tools, and currently there are too few types of GVs truly used in the treatment field, resulting in a narrow range of GVs particle sizes available for selection. The GVs cannot penetrate complex tissues and exert their effects, which greatly limits their application range. Therefore, the replenishment of GVs with new particle sizes is extremely important for future application expansion. At present, artificial intelligence is developing rapidly, and in the future, machine learning and other technologies may be used to manually design non-natural GVs structures, achieving purpose-oriented design and promoting the vigorous development of ultrasound contrast agents. In brief, as a nanomaterial, GVs may expand, even outside, the application scope in the fields of biology and medicine.

## Supplementary Information

Supplementary Material 1.

## Data Availability

Data sharing not applicable to this article as no datasets were generated or analyzed during the current study.
