# Supplementary material for: Excavation of acoustic nanostructures biosynthesis gene clusters by combinatorial strategy
Source: Adv Biotechnol (Singap). 2025 May 15;3(2):15. doi: 10.1007/s44307-025-00069-5 (PMC12081810; doi:10.1007/s44307-025-00069-5)
Supplement: Supplementary file 1 — Supplementary Material 1. [file 44307_2025_69_MOESM1_ESM.docx]

**Excavation of Acoustic Nanostructures Biosynthesis Gene Clusters by Combinatorial Strategy**

Wei Liu^1^, Tingting Liu^2^, Shenxi Huang^1^, Fei Yan^3^*, and Jian-Zhong Liu^1^*

*1 State Key Laboratory of Biocontrol, School of Life Sciences, Sun Yat-Sen University, Guangzhou 510275, China*

*2 Department of Ultrasound, The Second People’s Hospital of Shenzhen, The First Affiliated Hospital of Shenzhen University, Shenzhen 518061, China*

*3 CAS Key Laboratory of Quantitative Engineering Biology, Shenzhen Institute of Synthetic Biology, Shenzhen Institutes of Advanced Technology, Chinese Academy of Sciences, Shenzhen 518055, China*

Email:

Wei Liu: liuw283@mail2.sysu.edu.cn

Tingting Liu: ltingting949@gmail.com

Shen-Xi Huang: huangshx23@mail2.sysu.edu.cn

Fei Yan: fei.yan@siat.ac.cn

Jian-Zhong Liu: lssljz@mail.sysu.edu.cn

* Corresponding author: School of Life Science, Sun Yat-Sen University, Guangzhou 510275, P.R. China.

**Table S1:** The specific data of Flotation Assay.

|  |  | Structural genes | | | | | |
| --- | --- | --- | --- | --- | --- | --- | --- |
|  |  | StAf | StBm | StSc | StHs | StHC | StSe |
| Accessory genes | AcAf | 0.163±0.004 | 0.148±0.003 | 0.147±0.003 | 0.128±0.002 | 0.073±0.004 | 0.103±0.008 |
|  | AcBm | 0.369±0.008 | 0.243±0.009 | 0.213±0.004 | 0.215±0.014 | 0.235±0.006 | 0.364±0.035 |
|  | AcSc | 0.154±0.004 | 0.196±0.010 | 0.113±0.007 | 0.152±0.004 | 0.119±0.006 | 0.145±0.004 |
|  | AcHs | 0.168±0.003 | 0.157±0.004 | 0.192±0.004 | 0.195±0.003 | 0.164±0.003 | 0.108±0.007 |
|  | AcHC | 0.180±0.007 | 0.168±0.011 | 0.143±0.004 | 0.202±0.004 | 0.187±0.016 | 0.151±0.005 |

(mean OD_600_ ±SD, N=3)


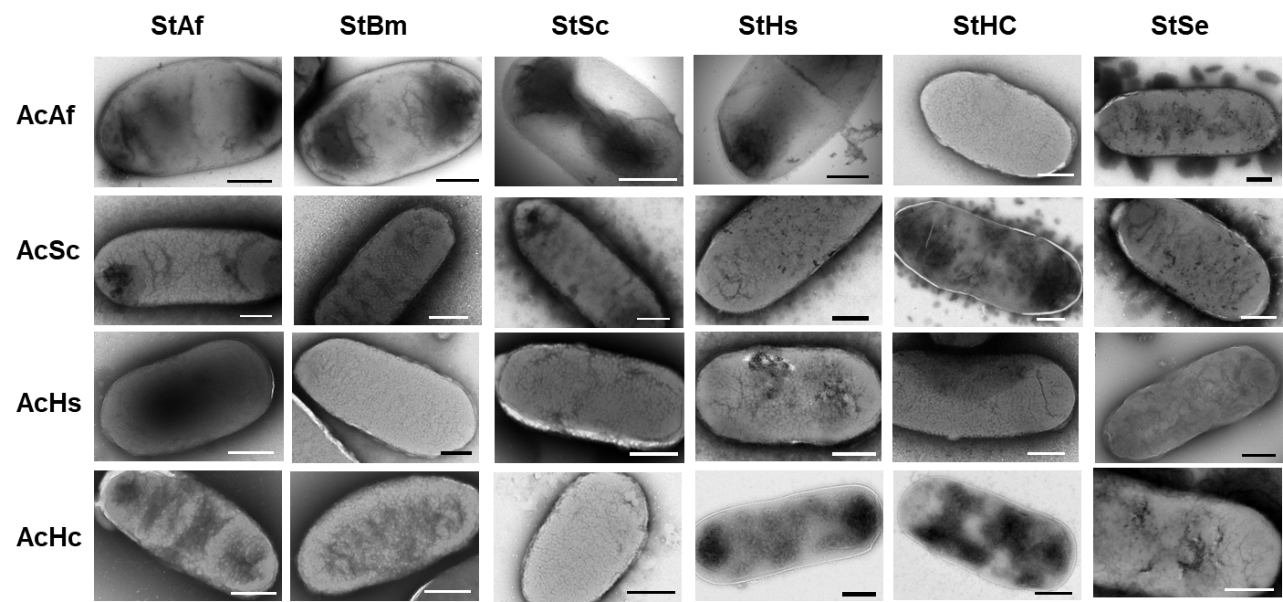


**Figure S1:** Partial TEM images of cells expressing hybridized BGCs. Scale bars, 500 nm.


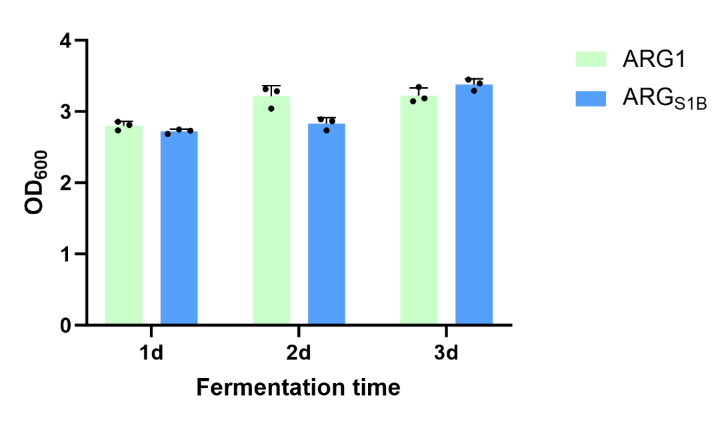


**Figure S2:** The optimization of fermentation time. Error bars representing mean ± SD for N = 3 replicates.


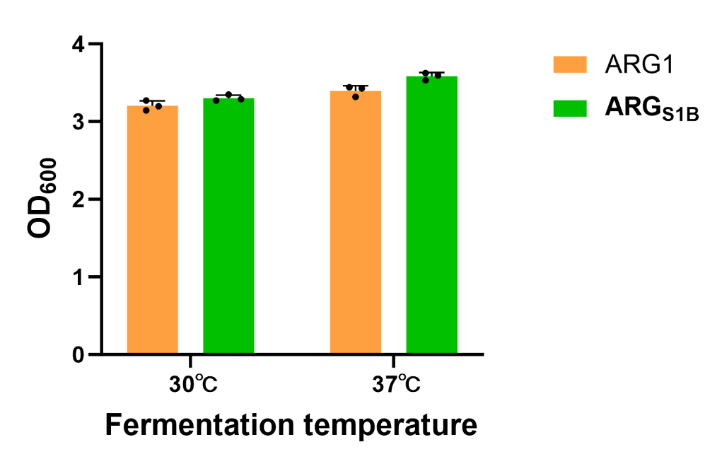


**Figure S3:** The optimization of fermentation temperature. Error bars representing mean ± SD for N = 3 replicates.


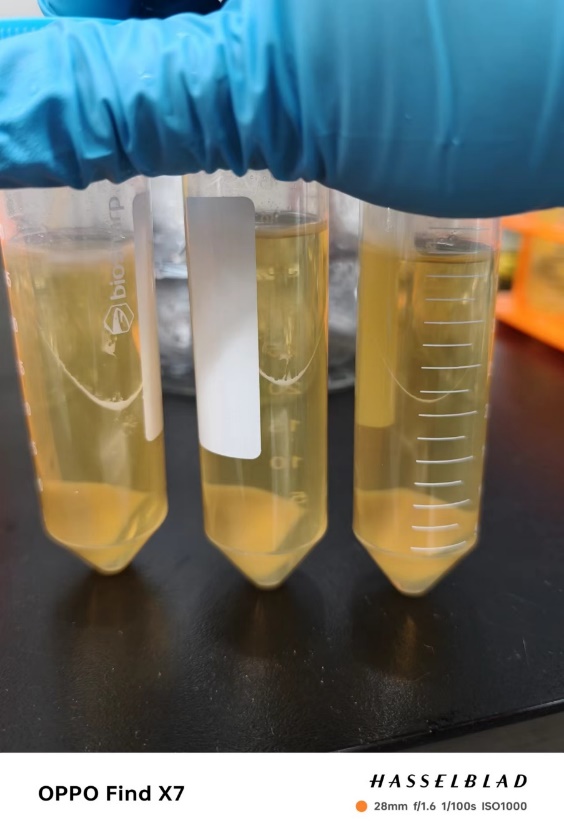


**Figure S4:** Improvement of GVs yield of ARG_S1B_ after multi optimization of fermentation conditions (from right to left).


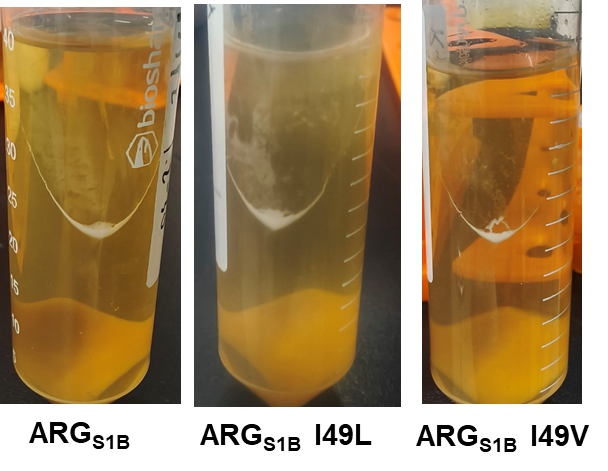


**Figure S5:** Floating bacterial cells of ARG_S1B_ and mutants after centrifugation (350g, 4hour).
